# Journalistic Notes

**Published:** 1891-10

**Authors:** 


### Journalistic Notes.

Dr. Bransford Lewis at Home. — In another column will be found the announcement of *The Fortnightly M. D.*, to be published at St. Louis by our talented foreign correspondent, who has just returned from an extended European tour, and is now in New York City, placing his advertising. Considerable interest and no end of curiosity has been aroused among medical men concerning the "guessing" of the title to the Doctor's journal, the weird emblem of which appears in connection with his advertisement. The result of the "guess" brings out some facts of peculiar interest to those who use the advertising pages of medical journals. Out of a combined circulation of about 150,000 in this and other journals, nearly 39,000 answers were received in three months, which goes to prove that even the smallest advertisements are read by western physicians.

The number guessing correctly, 6,422, was comparatively small, but Dr. Lewis can congratulate himself upon having on his list not only the *brightest thinkers* in the country, but doctors who will, no doubt, appreciate his efforts in the journalistic line. To those of our readers who missed on their guess, we would say, take another look at the design and see how simple it is (after you know). The letters "F. M. D." were no doubt easily read by those familiar with the deaf and dumb alphabet, while the moon's quarter (fortnightly) makes the interpretation complete. The instruments crossing under the monogram in the form of "X" are construed to mean "by," thus "Fortnightly M. D., by Bransford Lewis, St. Louis." We anxiously await the initial number, which is to appear January 1, 1892.— *Medical Herald, St. Joseph, Mo.*

---

A recent death from chloroform, reported in the *Indiana Medical Journal*, was attended by such disagreeable consequences to the physician, as to give point to the moral, that except in case of obstetrics or emergency, anesthetics should not be given by a physician without the presence of another physician.—*American Lancet.*

